# Very high high-density lipoprotein cholesterol may be associated with higher risk of cognitive impairment in older adults

**DOI:** 10.1186/s12937-024-00983-9

**Published:** 2024-07-17

**Authors:** Huifan Huang, Bin Yang, Renhe Yu, Wen Ouyang, Jianbin Tong, Yuan Le

**Affiliations:** 1 Department of Anesthesiology, the First Affiliated Hospital of Xiamen University, School of Medicine, Xiamen University, Xiamen, China; 2 Department of Anesthesiology, the Third Xiangya Hospital, Central South University, Changsha, China; 3 Department of Epidemiology and Health Statistics, Xiangya School of Public Health, Central South University, Changsha, China; 4 Hunan Province Key Laboratory of Brain Homeostasis, the Third Xiangya Hospital, Central South University, Changsha, China

**Keywords:** High-density lipoprotein cholesterol, Lipid metabolism, Cognitive function, Older adults, NHANES

## Abstract

**Background:**

Previous studies have shown that high-density lipoprotein cholesterol (HDL-C) levels are positively associated with cognitive function across a range of concentrations. However, recent studies have suggested that very high HDL-C levels may lead to poorer outcomes. Therefore, we aimed to investigate the relationship between different concentrations of HDL-C and cognitive impairment risk.

**Methods:**

We collected data from 3632 participants aged over 60 years from the U.S. National Health and Nutrition Examination Survey (NHANES) between 2011 and 2014 to assess the relationship between HDL-C and cognitive function. Cognitive function was evaluated with the Consortium to Establish a Registry for Alzheimer’s Disease (CERAD) test, the animal fluency test (AFT), and the digit symbol substitution test (DSST). We used restricted cubic spline models and logistic regression to examine the association between HDL-C and cognitive function.

**Results:**

A U-shaped was observed between HDL-C and cognitive outcomes, individuals with higher risk in those with both low and very high HDL-C levels compared with those with midrange values. Very high HDL-C levels (≥ 2.50 mmol/L) were associated with increased risk of cognitive impairment (OR = 2.19; 95% CI, 1.12–4.28) compared with those with HDL-C levels in the range of 1.50 to 1.99 mmol/L in older adults after adjustment for confounding factors. Interaction test demonstrated that relationship between very high HDL-C and the risk of cognitive impairment was not changed in different sex and race group (*P* for interaction > 0.05).

**Conclusions:**

Very high HDL-C levels were associated with an increased risk of cognitive impairment. HDL-C may not be a protective factor for maintaining brain health in older adults at very high levels.

## Introduction

In 2019, 700 million people in the world were over the age of 65, and by 2050, one out of six people will be older adults [1]. An increasing number of old people complain of cognitive decline [2], which is characterized by deficits in working memory, processing speed, and attention [3]. It significantly decreases the quality of life, imposes a disease burden on patients [4], and results in poor clinical outcomes [5–7].

At present, the development of cognitive decline involves a diversity of risk factors, such as low education level [8], comorbidities (i.e., metabolic diseases and depression) [9, 10] and health behaviors (i.e., cigarette dietary intake [11]). Among metabolic factors, abnormal lipid metabolism, including high-density lipoprotein cholesterol (HDL-C), low-density lipoprotein cholesterol (LDL-C), total cholesterol (TC) and triglycerides (TG), has been identified as an important factor in cognitive function [12, 13]. In particular, HDL-C, which is well known for its cardioprotective and anti-inflammatory functions [14], is strongly purported to have positive effects on cognitive function across a range of concentrations [12, 15–17]. But results form some recent studies indicated that HDL-C is associated with poorer outcomes at very high levels, such as cardiovascular events [18, 19], cerebrovascular disease [20], age-related macular degeneration [21], infections [22, 23], and mortality [18–24]. However, the relationship between very high concentrations of HDL-C and cognitive function remains unclear. We hypothesized that very high concentrations of HDL-C may lead to a higher risk of cognitive impairment. Accordingly, we conducted an analysis to explore the correlation between HDL-C and cognitive impairment in older individuals with a subsample of the National Health and Nutrition Examination Survey (NHANES) from 2011 to 2014.

## Materials and methods

### Study population

**Fig. 1:**
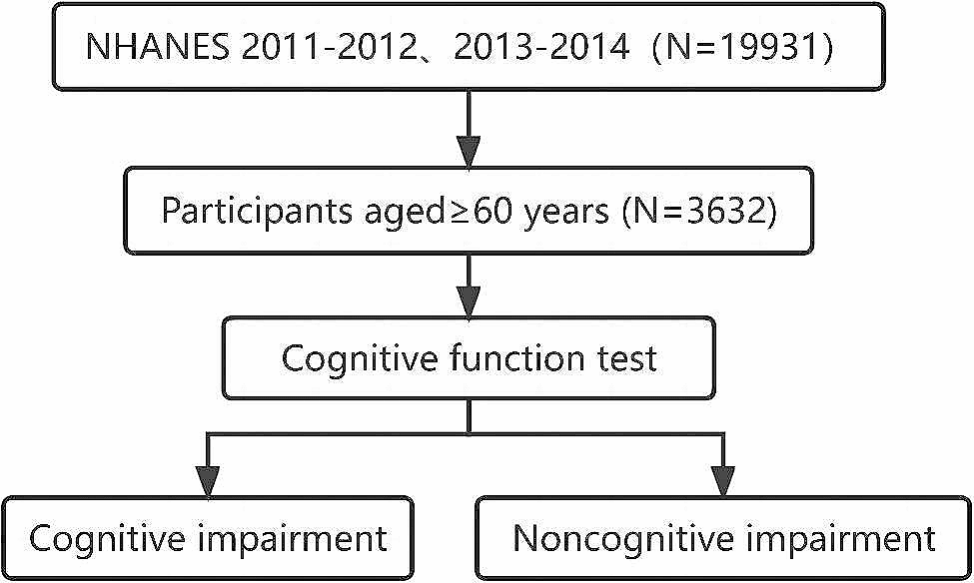
Flow chart for participants recruitment of this study, NHANES 2011–2014

The data were collected from the NHANES 2011–2014, which is a cross sectional study conducted by the National Centers for Health Statistics (NCHS). According to the inclusion criteria of our study, a total of 3632 participants aged over 60 years form the data of NHANES 2011–2014 were enrolled in the analyses (Fig. 1).

### Assessment of cognitive function

The cognitive function of participants was estimated using the following tests [25, 26]: (1) word learning and recall modules from the Consortium to Establish a Registry for Alzheimer’s Disease (CERAD), consisting of three consecutive learning trials, and a delayed recall. It assesses immediate and delayed learning ability for new verbal information (memory sub-domain); (2) the Animal Fluency test (AFT), which examines categorical verbal fluency, a component of executive function [27]; and (3) the Digit Symbol Substitution test (DSST), a performance module from the Wechsler Adult Intelligence Scale (WAIS III), relies on processing speed, sustained attention, and working memory [28]. These tests are widely used in cohort studies to assess memory, language, speech, and cognitive functions and to screen for risk factors [29–31].

To account for the ceiling and floor effect caused by a wide range of cognitive function in the elderly population, commonly known as the scale attenuation effect [32], we devised a method to create a global cognitive score. This was achieved by averaging the standardized scores of three cognitive tests: the CERAD, AFT, and DSST [33, 34]. Since there are no established cutoff points for these tests to determine low or normal cognitive function, we employed the lowest quartile in the study group to indicate cognitive impairment, consistent with methods previously published in literature [35].

### Measurement of serum lipid profiles

HDL-C, TC and TG levels in serum were measured by enzymatic assay [36–38]. LDL-C was calculated from the measured values of TC, HDL-C, and TG according to the Friedewald formula [38]. And normal range of serum lipids levels are as follows: HDL ≥ 1.04 mmol/L (40 mg/dL); LDL < 3.36 mmol/L (130 mg/dL); TC < 5.17 mmol/L (200 mg/dL); and TG < 3.88 mmol/L (150 mg/dL) [39].

### Covariates

We also obtained demographic characteristics, including sex, age, race, body mass index (BMI), educational level, hypertension, diabetes, smoking, alcohol consumption, lipid-lowering therapy and physical activity (PA) information. PA was categorized into low intensity and high intensity [40]. The chronic disease history (hypertension and diabetes) was self-reported based on the diagnosis of the physician. Individuals who had smoked more than 100 cigarettes in their life were classified as smokers. And alcohol consumption was classified as fewer than 12 drinks per year or more than 12 drinks per year.

### Statistical analysis

The data were downloaded from the NHANES database (2011–2014). The missing data on demographic information, cognitive function and serum lipid profiles of some people were handled with multiple imputation (MI) to minimize the bias of analysis results. We grouped HDL-C into five categories in accordance with prior study (< 1.0, 1.0 to 1.49, 1.5 to 1.99, 2.0 to 2.49, and ≥ 2.50 mmol/L) [41] and compared baseline characteristics between them. Continuous variables were represented as the mean ± SD and comparisons between participants with and without cognitive impairment were analyzed by t-tests; while categorical variables are represented as N (%) and the chi-square tests were used to compare the differences.

First, we entered HDL-C as a continuous variable and analyzed the possibility of nonlinearity between HDL-C and cognitive function using a restricted cubic spline model. The concentrations of HDL-C associated with the lowest risk of cognitive impairment was the concentrations with the lowest OR ratio in restricted cubic spline regression. Next, we taken HDL-C as a categorical variable and used the multivariable logistic regression to further study the relationship between HDL-C and cognitive function in different concentrations. Individuals with HDL-C levels between 1.50 and 1.99 mmol/L formed the reference. Model 1 was adjusted for sex, age, race, BMI, and educational level, and model 2 was further adjusted for physical activity, diabetes, hypertension, smoking, alcohol consumption, lipid-lowering therapy, LDL cholesterol and triglycerides. Finally, we performed interaction analysis to determine if the association between very high HDL-C and outcomes differed by sex and race after adjusting for aforementioned covariates.

All statistical analyses were performed with SPSS 26.0 and R 4.2.1, and *P* < 0.05 was considered statistically significant.

## Results

### Basic characteristics of participants

Table 1 lists the general characteristics of the participants in this study. A total of 3632 adults aged ≥ 60 years old were included in this study, of which 1760 (48.5%) were male and 1872 (51.5%) were female. Characteristics of participants by the HDL-C categories are summarized in the Table 1. Participants with higher levels of HDL-C tended to have lower body mass index, higher educational level, and to be more likely take part in high intensity physical activity and use lipid-lowering agents (Table 1).

**Table 1:** Characteristics of participants

Characteristics	HDL-C level (mmol/L)				
	< 1.00	1.00-1.49	1.50–1.99	2.00-2.49	≥ 2.50
**Toll participants**	518	1850	941	263	60
**Sex**,** n (%)**					
Male	382 (73.7)	967 (52.3)	321 (34.1)	71 (27.0)	19 (31.7)
Female	136 (26.3)	883 (47.7)	620 (65.9)	192 (73.0)	41 (68.3)
**Age**,** n (%)**					
60–69 years	269 (51.9)	959 (51.8)	460 (48.9)	132 (50.2)	28 (46.7)
70–79 years	164 (31.7)	542 (29.3)	261 (27.7)	77 (29.3)	25 (41.7)
≥ 80 years	85 (16.4)	349 (18.9)	220 (23.4)	54 (20.5)	7 (11.7)
**Race**,** n (%)**					
Mexican American	53 (10.2)	198 (10.7)	73 (7.8)	9 (3.4)	3 (5.0)
Other Hispanic	57 (11.0)	228 (12.3)	73 (7.8)	10 (3.8)	0 (0.0)
Non-Hispanic White	256 (49.4)	794 (42.9)	430 (45.7)	141 (53.6)	27 (45.0)
Non-Hispanic Black	97 (18.7)	428 (23.1)	249 (24.5)	69 (26.2)	28 (46.7)
Other race	55 (10.6)	202 (10.9)	116 (12.3)	34 (12.9)	2 (3.3)
**BMI**,** n (%)**					
<25 kg/m^2^	69 (13.3)	420 (22.7)	341 (36.2)	147 (55.9)	29 (48.3)
25 to < 30 kg/m^2^	207 (40.0)	655 (35.4)	316 (33.6)	70 (26.6)	23 (38.3)
≥ 30 kg/m^2^	242 (46.7)	775 (41.9)	284 (30.2)	46 (17.5)	8 (13.3)
**Educational level**,** n (%)**					
Less than high school	179 (34.6)	577 (31.2)	258 (27.4)	54 (20.5)	8 (13.3)
High school	136 (26.3)	428 (23.1)	206 (21.9)	54 (20.5)	15 (25.0)
College or higher	203 (39.2)	845 (45.7)	477 (50.7)	155 (58.9)	37 (61.7)
**Physical activity**,** n (%)**					
Low intensity	284 (54.8)	1009 (54.5)	477 (50.7)	115 (43.7)	23 (38.3)
High intensity	234 (45.2)	841 (45.5)	464 (49.3)	148 (56.3)	37 (61.7)
**Hypertension**,** n (%)**					
Yes	352 (68.0)	1191 (64.4)	582 (61.8)	132 (50.2)	34 (56.7)
No	166 (32.0)	659 (35.6)	359 (38.2)	131 (49.8)	26 (43.3)
**Diabetes**,** n (%)**					
Yes	189 (36.5)	510 (27.6)	145 (15.4)	23 (8.7)	6 (10.0)
No	329 (63.5)	1340 (72.4)	796 (84.6)	240 (91.3)	54 (90.0)
**Smoking**,** n (%)**					
Current	94 (18.1)	230 (12.4)	93 (9.9)	36 (13.7)	12 (20.0)
Never	213 (41.1)	911 (49.2)	530 (56.3)	131 (49.8)	32 (53.3)
Former	211 (40.7)	709 (38.3)	318 (33.8)	96 (36.5)	16 (26.7)
**Alcohol consumption**,** n (%)**					
Yes	349 (67.4)	1155 (62.4)	586 (62.3)	182 (69.2)	53 (88.3)
No	169 (32.6)	695 (37.6)	355 (37.7)	81 (30.8)	7 (11.7)
**Lipid-lowering therapy**,** n (%)**					
Yes	431 (83.2)	1542 (83.4)	789 (83.8)	214 (81.4)	46 (76.7)
No	87 (16.8)	308 (16.6)	152 (16.2)	49 (18.6)	14 (23.3)
**HDL-C (mmol/L)**	33.4 (3.9)	47.9 (5.3)	65.7 (5.3)	83.9 (5.2)	110.7 (16.4)
**LDL-C (mmol/L)**	97.8 (32.5)	104.2 (35.0)	103.8 (35.2)	101.0 (32.6)	101.1 (32.6)
**TG (mmol/L)**	206.5 (148.6)	173.4 (135.0)	145.9 (129.3)	135.6 (130.9)	103.8 (95.5)
**Cognitive impairment**,** n (%)**					
Yes	161 (31.1)	464 (25.1)	219 (23.3)	45 (17.1)	17 (28.3)
No	357 (68.9)	1386 (74.9)	722 (76.7)	218 (82.9)	43 (71.7)

BMI: body mass index; HDL-C: high-density lipoprotein cholesterol; LDL-C: low-density lipoprotein cholesterol; TG: triglyceride

### Restricted cubic spline regression analysis between HDL-C and cognitive impairment

**Fig. 2:**
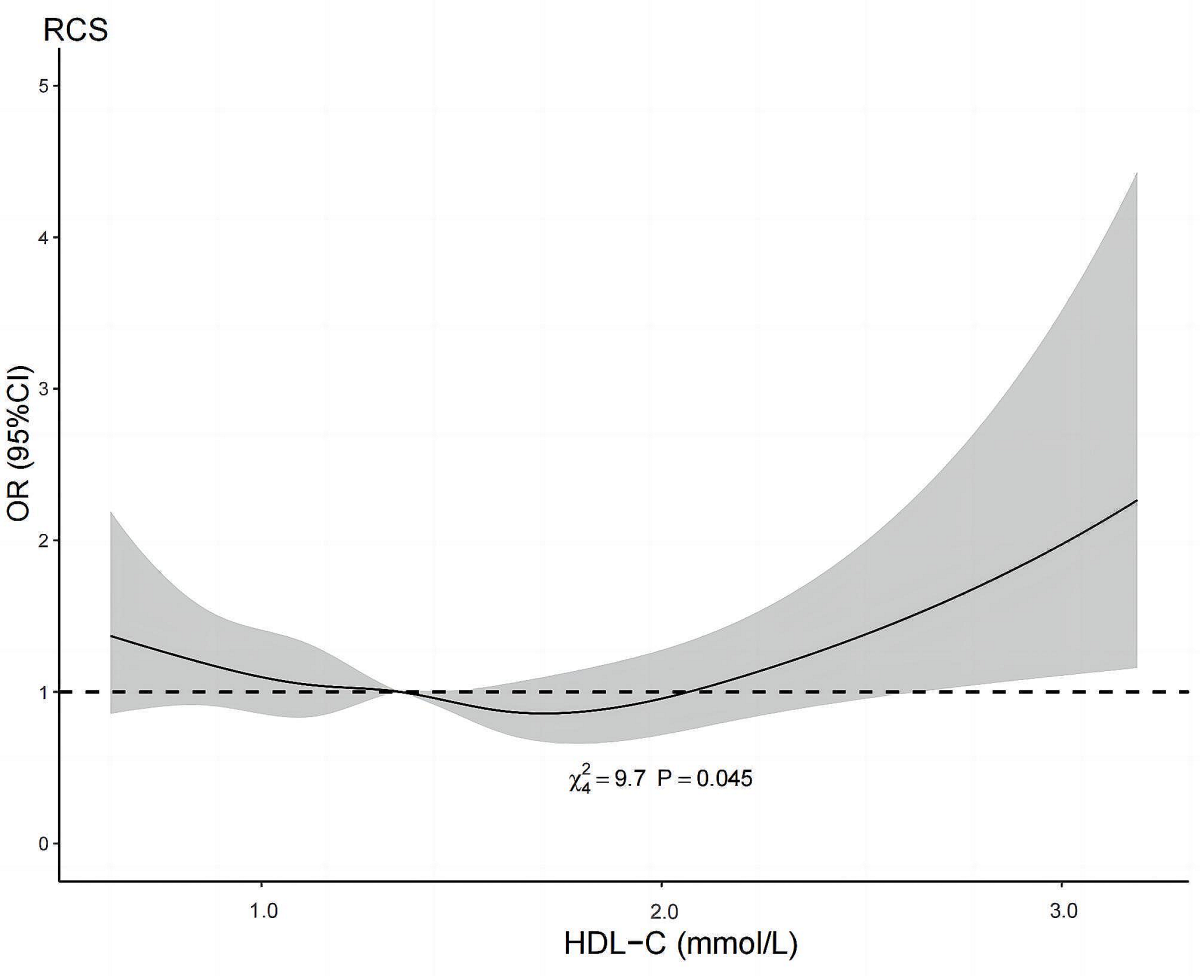
The odds ratio of cognitive impairment with HDL-C. Adjusted for age, sex, race, BMI, educational level, physical activity, diabetes, smoking, alcohol consumption, lipid-lowering therapy, LDL cholesterol and triglycerides. OR, odds ratio; CI, confidence interval

For the restricted cubic spline model analysis, we found a nonlinear curve relationship between HDL-C and cognitive function (*P* = 0.045, Fig. 2). The graph shown that the risk of cognitive impairment decreases with increasing concentrations of HDL-C, reaching a minimum risk of approximately 1.70 mmol/L and increasing thereafter. Individuals with lower or higher concentrations of HDL-C had a greater tendency to have poor cognitive outcome. The concentration of HDL-C associated with a lower risk of cognitive impairment was approximately 1.35 to < 2.05 mmol/L.

### Multivariable logistic regression analysis between HDL-C and cognitive impairment

As shown in Table 2, after full adjustment for covariates, compared with individuals with HDL-C in the range of 1.55 to 1.99 mmol/L, the OR for risk of cognitive impairment was 2.19 (95%Cl, 1.12–4.28) for individuals with concentrations of HDL-C ≥ 2.50 mmol/L in the model 2 (Table 2).

**Table 2:** Logistic regression of different concentrations of HDL-C and cognitive impairment

HDL-C (mmol/L)	Events (*n*)	%	Crude	Model 1	Model 2
< 1.00	161/518	31.1	1.49 (1.17, 1.89)*	1.54 (1.16, 2.05)*	1.43 (1.06, 1.92)*
1.00-1.49	464/1850	25.1	1.10 (0.92, 1.33)	1.10 (0.89, 1.36)	1.04 (0.84, 1.30)
1.50–1.99	219/941	23.3	Reference	Reference	Reference
2.00-2.49	45/263	17.1	0.68 (0.48, 0.97)*	0.80 (0.54, 1.19)	0.87 (0.58, 1.30)
≥ 2.50	17/60	28.3	1.30 (0.73, 2.33)	1.90 (0.99, 3.66)	2.19 (1.12, 4.28)*

Model 1: adjusted for age, sex, race, BMI and educational level;

Model 2: adjusted for age, sex, race, BMI, educational level, physical activity, diabetes, smoking, alcohol consumption, lipid-lowering therapy, LDL cholesterol and triglycerides; *P < 0.05

### Interaction analysis

**Fig. 3:**
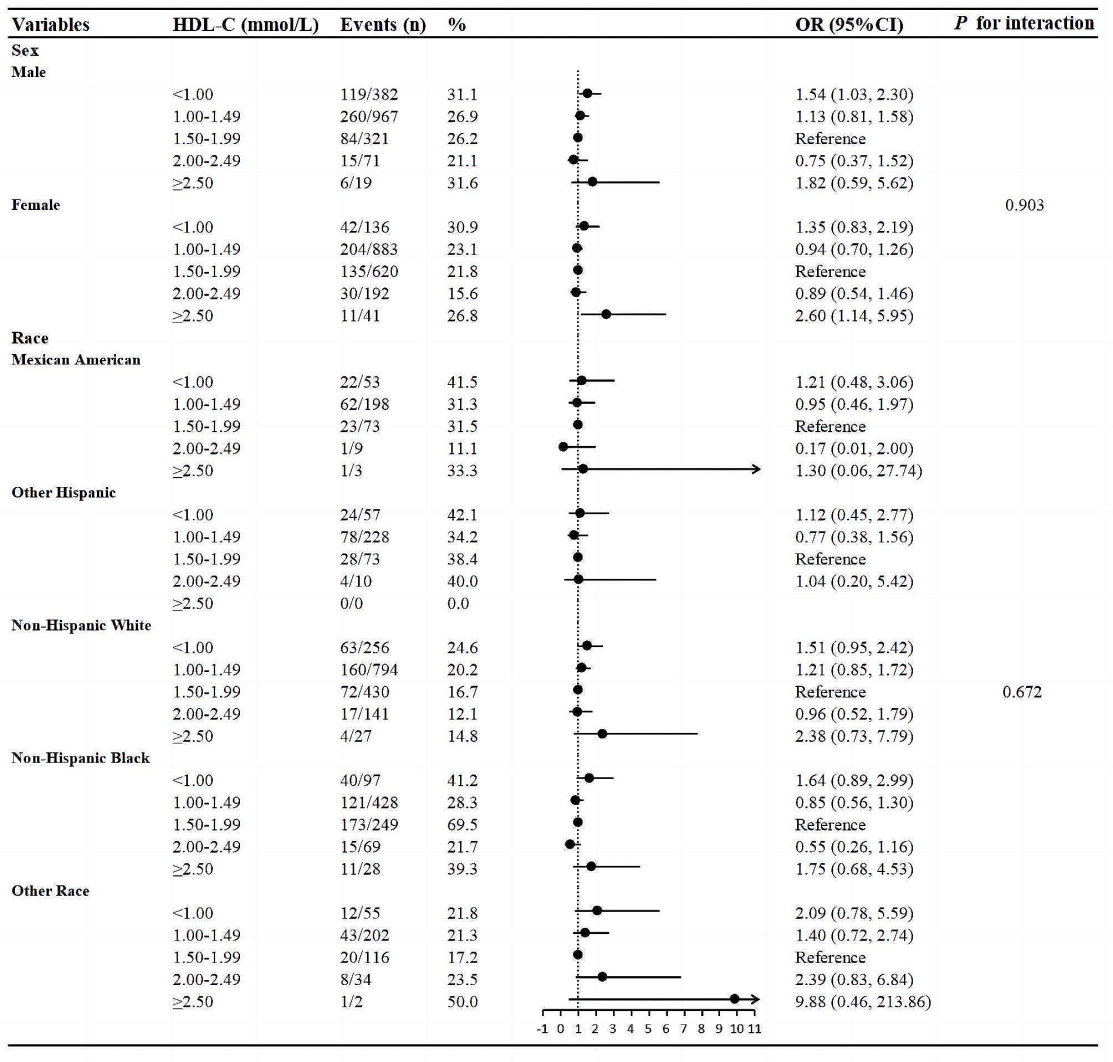
Forest plot of the relationship between HDL-C and cognitive impairment. Adjusted for age, BMI, educational level, physical activity, diabetes, smoking, alcohol consumption, lipid-lowering therapy, LDL cholesterol and triglycerides. OR, odds ratio; CI, confidence interval

Finally, we further conducted stratified analysis and interaction test of the relationship between HDL-C and cognitive impairment (Fig. 3). As results, there was no significant interaction with sex and race was observed in the group with HDL-C level greater than 2.50 mol/L for cognitive impairment (All *P* for interaction > 0.05), which indicated that the association between HDL-C and cognitive function was still stable in different sex and race subgroups.

## Discussion

Results of our study suggested that in older adults, compared with those with normal HDL-C levels (1.50–1.99 mmol/L), those with very high HDL-C levels (> 2.50 mmol/L) have a higher risk of poor cognitive outcome, independent of traditional risk factors. Our study indicated that high HDL-C is not always a protective factor for brain health. The protective effect of HDL-C may not appear at very high levels, instead of bringing increased risk.

Prior studies have imputed that higher HDL-C levels are associated with better cognitive function status compared with those with normal HDL-C levels [12, 15–17], while lower HDL-C levels are related to adverse outcomes [42]. The potential mechanism is that HDL-C is able to bind Aβ and prevent Aβ aggregation into amyloid [43, 44] and then improve the clearance of Aβ from the brain. In this way, the neurotoxicity of Aβ peptides could be decreased [45]. Furthermore, another reason may be the anti-inflammatory and antioxidant properties of apoA-I/HDL [46, 47]. These findings highlight the beneficial role of high HDL-C in maintaining good cognitive function during aging.

However, our data showed that, just as low concentrations of HDL-C can raise the risk of poor cognitive function in the population, too high concentrations of HDL-C (≥ 2.50 mmol/L) are also unfavorable to maintain better cognitive status. Recently, some studies have begun to investigate the correlation between very high concentrations of HDL-C and adverse outcomes. Results from two large cohort study concluded that people with too high concentrations of HDL-C (> 2.07 mmol/L) have a higher frequency of cardiovascular-related risk and mortality [18, 19]. Also, studies on cerebrovascular disease [20], AMD [21], infections [22, 23], and mortality [18–24] found similar results. European guidelines highlight that HDL-C should not be used as a risk measure when HDL-C levels exceed 90 mg/L (2.3 mmol/L) [48]. In addition, evidence from multiple randomized clinical trials shown that elevating HDL therapies had failed to significantly reduce adverse health outcome [49–51].

A study focus on people with Parkinson’s disease found that higher serum HDL-C were associated with poorer cognitive function in females [52]. Another, a large cohort study supported our findings, as they observed that HDL-C above 1.70 mmol/L (65 mg/dL) was significantly related to an increased rapid decline in overall cognition [53]. However, their study was based on a Chinese population with an age range of 50–70 years, whereas analysis of our study contained a much wider range of ages (including an older population over 70 years old) and races (it was multiethnic).

The potential underlying mechanisms by which very high HDL-C levels may be associated with higher cognitive impairment are still not clear. One possible explanation is that very high concentrations are usually caused by genetic variation of HDL-C, such as CETP, LIPC, and SCARB1 [54–57]. For example, it was shown that HDL-C with certain mutations may be correlate with a higher frequency of adverse cardiovascular [55–57] and cerebrovascular outcomes [58]. Another reason may be that the functional properties of HDL-C are impaired in individuals with extreme high HDL-C levels [59]. Generally, mature HDL-C is capable of promoting cholesterol efflux mediated by ABCG1, SR-BI and probably other mechanisms [60]. However, dysfunctional HDL-C reduced the efflux of cholesterol from macrophages by the ABCG1 and activated pro-inflammatory signaling [61]. And very high HDL-C levels (> 2.2 mmol/L) could promote oxidative stress reaction and inflammatory response [62]. These suggest that extremely high concentrations of HDL-C may be related to the adverse cognitive outcomes. However, the exact mechanism needs further investigation.

Our study has some limitations. First, concerning that concentrations of HDL-C varies among different sex (females on average have higher concentrations of HDL-C than males) and the effect of HDL-C may differ by race [63], we performed stratified analysis to study the relationship between HDL-C and cognitive impairment by sex and race. However, numbers in extreme high HDL-C groups (≥ 2.50 mmol/L) were small, limiting statistical power, which may account for the *P* value not achieve statistical significance in groups ≥ 2.50 mmol/L in subgroups. But interaction test shown that results of our study would not affect by sex and race (*P* for interaction > 0.05). So we still believe that our results are reliable. Second, as this is an observational cross-sectional study, it is unable to determine whether the association between HDL-C and cognitive impairment is causal. Additionally, the population of our study focuses on people over 60 years old, whether the relationship between HDL-C and cognitive function found in our study can be extended to other age groups is unknown and requires further exploration in the future.

## Conclusions

In conclusion, our study indicated that very high levels of HDL-C may be associated with a greater risk of cognitive impairment. At present, HDL-C may not a good target for drug-based treatment. In the future, more analyses is needed to confirm the association between HDL-C and cognitive impairment in old people and to uncover the exact mechanism linking them.

## Data Availability

The publicly available data can be obtained here: https://www.cdc.gov/nchs/nhanes/index.htm.
